# Supplementary material for: The interactome of intact mitochondria by cross-linking mass spectrometry provides evidence for coexisting respiratory supercomplexes
Source: Mol Cell Proteomics. 2017 Dec 8;17(2):216–32. doi: 10.1074/mcp.RA117.000470 (PMC5795388; doi:10.1074/mcp.RA117.000470)
Supplement: Supplemental Data [file supp_RA117.000470_133922_0_supp_23978_fzffwf.zip › spectra_annotation/mito_spectra_annotation/119-1-1-1-143-1.pdf]

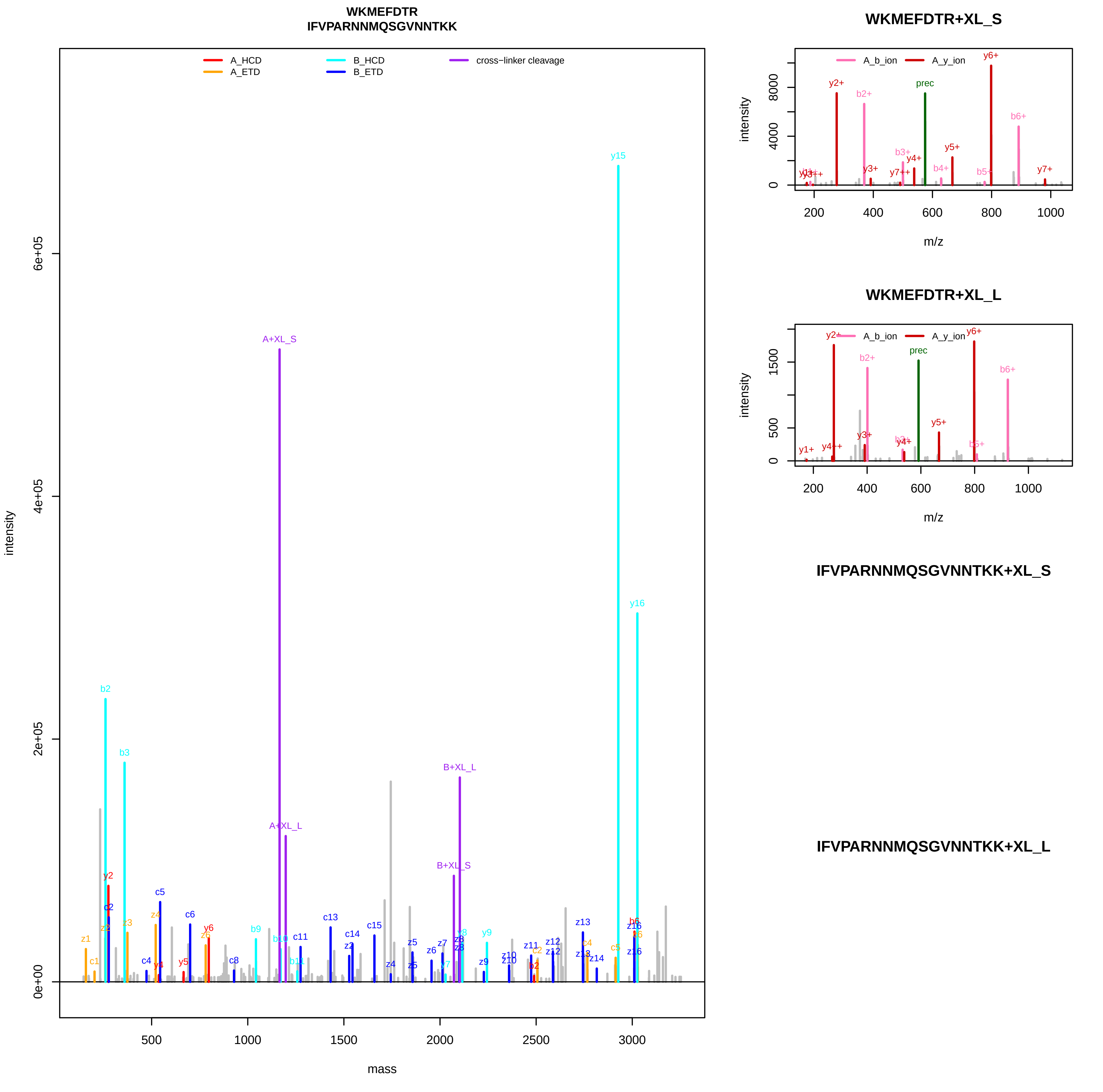
WKMEFDTR
IFVPARNNMQSGVNNTKK
A_HCD
A_ETD
B_HCD
B_ETD
cross−linker cleavage
intensity
mass
0e+00
2e+05
4e+05
6e+05
500
1000
1500
2000
2500
3000
y15
y16
A+XL_S
A+XL_L
B+XL_L
B+XL_S
b2
b3
y2
c5
c6
c13
c15
z13
WKMEFDTR+XL_S
A_b_ion
A_y_ion
prec
y6+
y2+
b2+
b6+
y5+
y4+
y3+
b3+
b4+
b5+
y7+
y7++
0
4000
8000
200
400
600
800
1000
m/z
WKMEFDTR+XL_L
0
500
1500
y1+
y4++
IFVPARNNMQSGVNNTKK+XL_S
IFVPARNNMQSGVNNTKK+XL_L
